# Genes Expressed in Grapevine Leaves Reveal Latent Wood Infection by the Fungal Pathogen *Neofusicoccum parvum*

**DOI:** 10.1371/journal.pone.0121828

**Published:** 2015-03-23

**Authors:** Stefan Czemmel, Erin R. Galarneau, Renaud Travadon, Andrew J. McElrone, Grant R. Cramer, Kendra Baumgartner

**Affiliations:** 1 Department of Biochemistry and Molecular Biology, University of Nevada, Reno, Nevada, United States of America; 2 United States Department of Agriculture-Agricultural Research Service, Davis, California, United States of America; 3 Department of Plant Pathology, University of California Davis, Davis, California, United States of America; University of Minho, PORTUGAL

## Abstract

Some pathogenic species of the Botryosphaeriaceae have a latent phase, colonizing woody tissues while perennial hosts show no apparent symptoms until conditions for disease development become favorable. Detection of these pathogens is often limited to the later pathogenic phase. The latent phase is poorly characterized, despite the need for non-destructive detection tools and effective quarantine strategies, which would benefit from identification of host-based markers in leaves. *Neofusicoccum parvum* infects the wood of grapevines and other horticultural crops, killing the fruit-bearing shoots. We used light microscopy and high-resolution computed tomography (HRCT) to examine the spatio-temporal relationship between pathogen colonization and anatomical changes in stem sections. To identify differentially-expressed grape genes, leaves from inoculated and non-inoculated plants were examined using RNA-Seq. The latent phase occurred between 0 and 1.5 months post-inoculation (MPI), during which time the pathogen did not spread significantly beyond the inoculation site nor were there differences in lesion lengths between inoculated and non-inoculated plants. The pathogenic phase occurred between 1.5 and 2 MPI, when recovery beyond the inoculation site increased and lesion lengths of inoculated plants tripled. By 2 MPI, inoculated plants also had decreased starch content in xylem fibers and rays, and increased levels of gel-occluded xylem vessels, the latter of which HRCT revealed at a higher frequency than microscopy. RNA-Seq and screening of 21 grape expression datasets identified 20 candidate genes that were transcriptionally-activated by infection during the latent phase, and confirmed that the four best candidates (galactinol synthase, abscisic acid-induced wheat plasma membrane polypeptide-19 ortholog, embryonic cell protein 63, BURP domain-containing protein) were not affected by a range of common foliar and wood pathogens or abiotic stresses. Assuming such host responses are consistent among cultivars, and do not cross react with other trunk/foliar pathogens, these grape genes may serve as host-based markers of the latent phase of *N*. *parvum* infection.

## Introduction

Interactions between plants and fungi are diverse, ranging from commensal and mutualistic to pathogenic. During pathogenesis, some fungi have a latent phase, co-existing for some time with their host plants, which show no apparent symptoms of disease at this time. Eventually, however, there is a ‘switch’ to the pathogenic phase, presumably when the environment and/or the host tissue becomes favorable for disease development. These latent pathogens can, hence, be distinguished from endophytic fungi, which can be defined as causing no disease symptoms throughout their association with their hosts [1].

Early detection of plant pathogens with a long, latent phase is a challenge for diagnosticians, depending on the type of plant tissue colonized during this phase. In the case of latent colonization of buds, flowers, or fruit, such tissues are conveniently sampled from a branch and the pathogens can then be cultured for detection, as for Panicle blight of pistachio caused by *Botryosphaeria dothidea* [2]. However, for pathogens that only colonize woody roots (e.g., Armillaria root rot caused by *Armillaria mellea*) or the trunk of a tree (e.g., Oak wilt caused by *Ceratocystis fagacearum*), sampling such tissues is destructive, especially if a symptomless plant is free of infection. For such pathogens, leaves could constitute a more convenient, non-destructive sample.

Some members of the fungal family Botryosphaeriaceae (Botryosphaeriales, Ascomycetes) are the most aggressive pathogens among the fungal communities that colonize the woody tissues of perennial hosts. Some Botryosphaeriaceae cause diseases of economically-important hosts, including timber trees (Sphaeropsis blight of pine) and horticultural crops (Lower limb dieback of almond). For example, *Neofusicoccum parvum* is known as an aggressive pathogen of a wide range of hosts [3]. It is one of the most aggressive causal agents of Botryosphaeria dieback of grapevine [4], which significantly limits vineyard productivity in all major grape-growing regions of the world [5]. Botryosphaeria dieback is categorized as a trunk disease, along with Esca, Eutypa dieback, and Phomopsis dieback, the causal agents of which are other ascomycete fungi. Trunk diseases are so named because a necrotic wood canker/lesion develops in the permanent woody structure, which includes the trunk, cordon, and spurs. The disease causes a ‘dieback’ as buds and shoots die distal to the wood canker, thereby limiting fruit production. The delay between infection by *N*. *parvum* and the appearance of dieback is considered a latent phase, a characteristic shared by many pathogenic species of Botryosphaeriaceae [3].

Although *N*. *parvum* does not reside in leaves, these tissues have been shown to undergo biochemical and molecular changes in response to infection by other trunk pathogens, such as the Esca pathogens *T*. *minima* and *P*. *chlamydospora* [6–9]. Detection of pathogen-based markers in leaves, such as secondary metabolites and toxins, may not provide sufficient resolution during the latent phase. Indeed, phytotoxic compounds produced by *T*. *minima* and *P*. *chlamydospora* are translocated to leaves, but are undetectable when leaves are asymptomatic [10]. Host-based markers may instead be more promising for detecting latent infections.

Detecting the latent phase of infection is critical to farmers who grow long-lived, woody crops. They must bear the long-term costs of unknowingly maintaining diseased plants that are doomed to low productivity. It is also critical to regulatory agencies in preventing pathogens, which may be present in asymptomatic plants in the latent phase of infection, from being introducted to new areas. Accordingly, a focus on identifying host-based markers of infection may advance the development of detection tools for vascular pathogens. Transcriptome profiling is a common approach to identify differentially-expressed genes (DEGs) associated with the host response to infection or identification of host genes associated with disease resistance. It is becoming more common for diseases of horticultural crops (e.g., Huanglongbing of citrus [11], Eutypa dieback of grapevine [12]), for which host genomes have been recently sequenced. In this study, we extended this approach to identify DEGs as putative host-based markers of infection. The long-term goal is to develop a field detection tool for asymptomatic leaves that are sampled in the latent phase of wood infection. In the short term, the goal is to identify DEGs that are specific to the host response to latent infection, in a controlled inoculation experiment in the glasshouse.

We defined the latent phase of infection of *Vitis vinifera* cv. ‘Cabernet Sauvignon’ inoculated with *N*. *parvum*. In the glasshouse, symptom expression signifies the pathogenic phase, which occurs after 2 to 3 months, when shoots die back and wood lesions can reach 15 cm in the stems of susceptible cultivars [13]. To ‘capture’ the latent phase, we examined plants for 2 months. From sections of woody stems, light microscopy was used to track pathogen spread and to identify plant anatomical responses to infection, which have not previously been reported in the pathogenesis of *N*. *parvum*, but have been examined with respect to unrelated ascomycete fungi that cause other trunk diseases (e.g., Eutypa dieback [14], Esca [15]). High resolution computed tomography (HRCT) was used to visualize xylem-vessel occlusions within the stems of intact plants [16]. In order to identify DEGs in response to *N*. *parvum*, but not wounding, genome-wide expression analysis was performed on asymptomatic leaves of inoculated-wounded (IW) and non-inoculated-wounded (NIW) plants, using RNA-Seq and validative qPCR. By screening 21 publicly-available microarray datasets, we identified a transcriptome signature, which did not respond to a range of abiotic stresses or common foliar and trunk diseases examined in other grape studies.

## Materials and Methods

### Inoculation of woody stems

The experiment was conducted in a glasshouse on potted Cabernet Sauvignon plants, propagated in 10 × 10-cm pots from green cuttings 12 months before inoculation [13]. Four sets of plants were inoculated at two-week intervals for 2 months. In this way, the 1^st^ set of plants represented the longest incubation period, 2 months post-inoculation (MPI), and the 4^th^ set of plants represented the shortest incubation period, 0.5 MPI. A power drill was used to wound the woody stem (2 × 3 mm) at 1 cm below the uppermost node, in a total of 88 plants. For IW plants, 20 μl of homogenized, 3-d liquid culture of *N*. *parvum* was pipetted into the wound that was afterwards sealed with Vaseline (Unilever, Greenwich, CT) and Parafilm (American National Can, Chicago, IL), as detailed previously [13]. NIW plants were ‘mock-inoculated’ with 20 μl of sterile Potato Dextrose Broth (PDB; Difco Laboratories). Five non-inoculated, non-wounded (NINW) plants were ‘untouched’ throughout the experiment; only young, immature leaves were harvested for RNA extraction. Plants were arranged in a completely randomized design with 14 h light per day.

Within 10 min of inoculation treatment, the 3^rd^ leaf of each plant was collected with flame-sterilized forceps, frozen in liquid nitrogen, and stored at -80°C for RNA extraction. Leaf samples from IW and NIW plants served as the 1^st^ sampling point, IW-0 MPI and NIW-0 MPI, respectively, for controls in RNA-Seq analyses. These controls were necessary to distinguish transcriptomic responses to infection and wounding. Leaf samples from NINW plants collected at this same time, NINW-0 MPI, served as the 1^st^ sampling point for controls in culture-based recovery and microscopy. These controls were necessary to distinguish fungal colonization and anatomical responses to infection and wounding. Stems of IW-0 MPI and NIW-0 MPI plants were not examined by culture-based recovery, HRCT, or light microscopy because within 10 min of inoculation we did not expect to detect measureable changes in pathogen growth or wood anatomy. Also on each inoculation date, leaves were collected from all plants from past inoculation dates, to provide leaf RNA samples for repeated measures of gene expression at 0.5, 1, 1.5, and 2 MPI.

Stem analyses were carried out on separate subsets of plants for either culture-based recovery of the pathogen [six IW and NIW plants per incubation period from 0.5 to 2 MPI and six NINW–0 MPI plants (54 total plants)] or HRCT [five IW and NIW plants per incubation period from 0.5 to 2 MPI and five NINW–0 MPI plants (45 total plants)]. Steps for recovery were as follows: bark was scraped off the stem, which was surface-sterilized in 1% sodium hypochlorite, and then cut longitudinally to measure lesion length [13]. Four pieces of wood were collected from the inoculation site (0 cm) and from 2 cm above and below (2 and -2 cm, respectively), surface-sterilized, and plated on potato-dextrose agar (PDA) amended with tetracycline (1 mg L^-1^). Positive recovery of the pathogen was based on colony morphology after 5 d growth on PDA.

#### High resolution computed tomography (HRCT)

HRCT was used to visualize xylem-vessel occlusions (tyloses, gels) within the stems of intact plants [16]. During scanning, the plant was rotated in 0.25° increments over 180°, yielding 720 two-dimensional projection images, which were reconstructed into three-dimensional projections displaying a vertical perspective of the stem, starting from the surface of the bark and moving in towards the pith (Octopus 8.3, Institute for Nuclear Sciences, Ghent, Belgium). At 2 MPI, five IW and NIW plants per incubation period and five NINW plants (45 total plants) were scanned in the 15 keV synchotron X-ray beam, during a 24-hour period (Advanced Light Source, Lawrence Berkeley National Lab, Berkeley, CA [17]). Within 12 h of scanning, stem segments (~8 cm in length × ~8 mm in diameter) from each plant were fixed in 4% gluteraldehyde in 1× phosphate-buffer solution (PBS) for microscopy.

#### Microscopy

Of the plants that were scanned for HRCT, three IW and NIW plants per incubation period from 0.5 to 2 MPI and three NINW plants (27 total plants) were prepared for examination by light microscopy. From each stem fixed in 4% gluteraldehyde, we separated out three 1-cm-long segments spanning the 2, 0, and -2 cm stem locations. These stem segments were then dehydrated in an ethanol series to 95% ethanol, infiltrated and embedded in LR White Resin (medium grade, Electron Microscopy Sciences, Hatfield, PA), and heat polymerized at 53°C for 6 h. The embedded stem segments were transverse-sectioned to 10-μm sections on a sliding microtome (American Optical Company, Buffalo, NY) with a tungsten carbide knife (Delaware Diamond Knives, Wilmington, DE).

Fungal colonization was quantified at 0 cm from six 10-μm sections per plant (27 plants × six sections per plant = 162 total sections). Because *N*. *parvum* is pigmented, its mature hyphae are visible in tissues of IW plants. Immature hyphae are not possible to distinguish from contaminating fungi, however, and so fungal colonization may have included hyphae of contaminating fungi. Fungal colonization was quantified at 200× (Leica DM5000B, Leica Microsystems, Heidelberg, GmbH) after staining with Pianeze IIIb [18]. Within each section, the presence of hyphae was noted in each ray zone, which represents xylem contained by two adjacent multiseriate rays and bordered by the cambium and the pith (approximately 38 to 41 ray zones per stem section). The proportion of colonized ray zones per section was assessed visually on a 0 to 100% ordinal scale (0–25, 26–50, 51–75, or 76–100%), and was then averaged among the six sections per plant. Homogeneity of variance across treatments was evaluated using Levene’s test. Non-parametric analysis performed with PROC MIXED in SAS 9.2 (SAS Institute, Cary, NC) [19] was used to determine the effects of inoculation treatment (IW, NIW), incubation period (0.5, 1.0, 1.5, 2.0 MPI), and their interactions on fungal colonization. NINW plants represented the 0 MPI incubation period. Relative treatment effects and 95% confidence intervals were calculated from PROC MIXED lsmeans, using the LD_CI macro [20].

**Fig 1:**
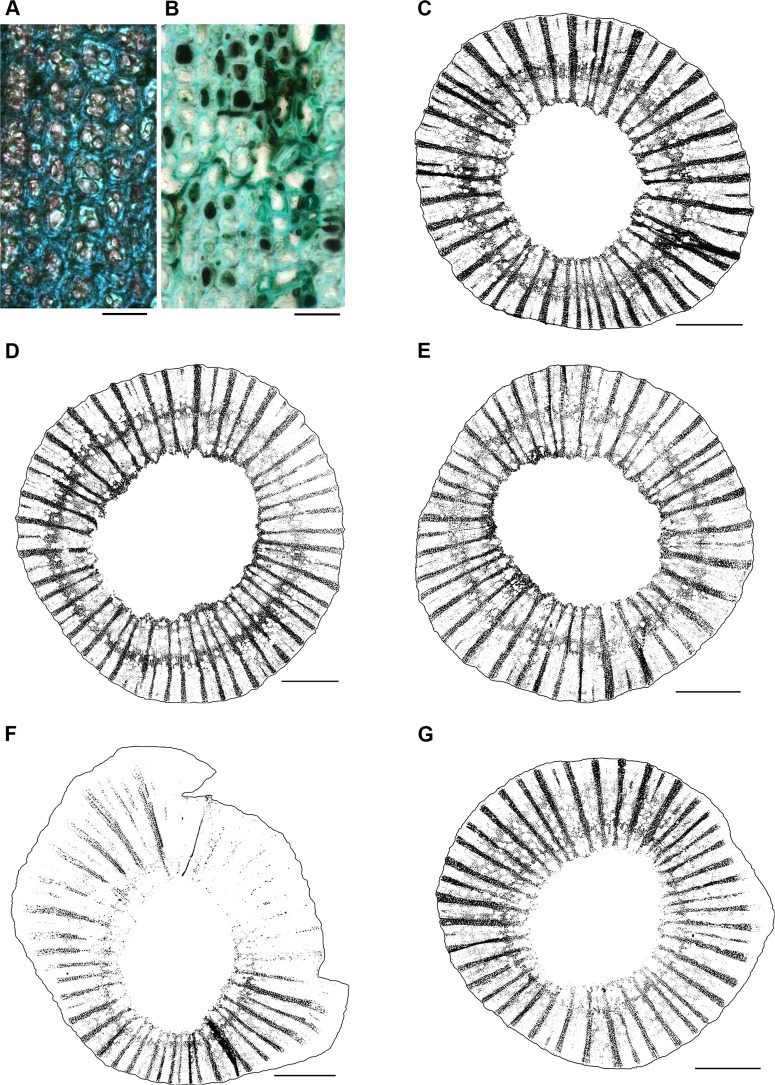
High levels of fungal colonization of the xylem fibers and rays were associated with starch depletion, especially at 2.0 MPI. (A) Xylem fibers of NIW plants contained starch (pink granules) and no hyphae, whereas those of IW plants (B) were devoid of starch, hence the drastic difference in stain absorption, and instead contained the darkly-pigmented hyphae of *N*. *parvum* (Bar, 25 μm). Starch content was quantified from I_2_/KI—stained sections at 0 cm (binary images shown here) for (C) NINW, (D) IW-0.5 MPI, (E) NIW-0.5 MPI, (F) IW-2.0 MPI, and (G) NIW-2.0 MPI. (C-G) Starch-filled fibers and rays are black; starch-deficient cells are white. Bar, 1 mm.

Starch granule content of the xylem fibers and rays was quantified using stereoscope light microscopy of I_2_/KI—stained, 10-μm sections from 0 cm (27 plants **×** three sections per plant = 81 total sections). Approximately 12 overlapping images per section (47.5×; Leica M205C stereoscope, Leica Microsystems) were merged together (Photoshop CS5, Adobe Systems Incorporated, San Jose, CA USA), then all but the xylem was cropped away (GIMP, v2.8.6, Free Software Foundation, Boston, MA USA). The resulting color image was converted to a binary image (FIJI v1.48, National Institutes of Health, USA) (Fig. 1). The area occupied by black pixels (% stem area) was averaged across three sections per plant.

Xylem vessel occlusions (tyloses and gels) were quantified at 2, 0, and -2 cm stem locations. From each location, three 10-μm sections (243 sections total) were examined. Xylem vessel occlusions were quantified within five 1.22 mm-diameter non-overlapping areas per section, then averaged across the five areas per section, and finally averaged among the three sections per location [21]. ANOVA was used to determine the effects of inoculation treatment (IW, NIW), incubation period (0.5, 1.0, 1.5, 2.0 MPI), stem location (2, 0, -2 cm), and their interactions on starch content and xylem vessel occlusions. NINW plants represented the 0 MPI incubation period. Prior to ANOVA, homogeneity of variance across treatments was evaluated using Levene’s test. ANOVAs were performed using the MIXED procedure in SAS, with all factors treated as fixed effects. For significant effects (*F* values with *P* <0.05), means were compared by Tukey’s tests. Location was treated as a repeated measure for xylem vessel occlusions because we anticipate correlations among measurements taken from the same stem. To satisfy the assumption of homogeneity of variance, square root transformations were applied to partial gels, partial tyloses, and full tyloses.

### RNA-Seq

Microscopy and HRCT were used to identify incubation periods that encompassed the latent phase of infection. Then leaf samples were selected for RNA-Seq. Three biological replicates, which contained a mixture of young, developing leaves (3^rd^ leaf counted from the shoot tip) from three plants, were used to compare IW and NIW for the 0 MPI incubation period versus a pooled sample of RNA extracted from leaves from three plants per incubation period for 0.5, 1, and 1.5 MPI. This gave 12 samples total in the following treatment-time combinations: IW–0 MPI, IW–0.5–1.5 MPI, NIW–0 MPI, and NIW–0.5–1.5 MPI. Total RNA was isolated using a rapid CTAB-based extraction method [22]. DNA was removed by RNase-Free DNase (Qiagen) digestion in solution and then purified using RNAeasy Mini Kit (Qiagen). Primers for leucoanthocyanidin reductase-1 were used to check RNA preparations for genomic DNA contamination [23] (S1 Table). RNA quality was analyzed using Experion RNA StdSens Chips (BioRad) to verify intact ribosomal bands [rRNA ratio (25s/18s) = 1.8–2.0] and RNA Quality Indicator (RQI) values >8. RNA-Seq libraries were prepared from 2 μg of RNA of either non-mixed (0 MPI) or mixed (0.5–1.5 MPI, 0.66 μg per RNA) at the DNA Technologies Core, University of California, Davis (http://dnatech.genomecenter.ucdavis.edu/wp-content/uploads/2013/11/TruSeq_RNA_Sample_Prep_Sept2012.pdf). All libraries were pooled and sequenced on two lanes of the same flow cell of an Illumina HiSeq 2500 to produce single-end 50-bp reads.

#### Read mapping, assembly, and differential-expression analysis

Raw Fastq files were quality-controlled with FastQC (http://www.bioinformatics.babraham.ac.uk/projects/fastqc/). Reads were aligned onto the grape genome (IGGP_12x, INSDC Assembly GCA_000003745.2, Jun 2011, downloaded from EnsemblPlants October 2013) using Tophat v2.0.10 [24, 25] with default mapping options, but using Bowtie1 as the aligner. The Tophat ready GFF3 file contained 29 927 predicted grape mRNA sequences. A matrix consisting of raw read counts for mRNA features in each sample was generated (HTSeq v0.5.4p5 [26]). Differential expression was then determined based on DESeq v1.10 for a multifactorial experiment [27]. To determine inference, general linear models were fitted to each gene based on the comparison of a full model (the potential influence of inoculation and incubation period) to a reduced model (the influence of incubation period only). Raw read Fastq data, raw read counts, and DESeq normalized read counts are deposited together with the experimental design of the RNA-Seq approach at Gene Expression Omnibus (GEO) at the National Center for Biotechnology Information (NCBI, http://www.ncbi.nlm.nih.gov/geo/) under accession GSE58653.

#### Clustering and GO analysis

The R package Hopach (v2.18.0) was used to partition genes into expression clusters [28]. GO analysis was performed using the BiNGO tool in Cytoscape v2.6 [29] on GO information downloaded from EnsemblPlants on April 2014, which annotated 18 657 of 29 971 genes. To retrieve the most informative terms, GO term relations were visualized using AmiGO, and the most finite terms were extracted. Over-represented categories were identified using a hypergeometric test with a significance threshold of 0.05 for gene clusters, after Benjamini and Hochberg False Discovery Rate (FDR) correction.

#### Primer design, cDNA synthesis, and qPCR analysis

Primers for candidate genes (S1 Table) were designed using NCBI Primer-BLAST [30] on consensus sequences derived from alignments of combined RNA-Seq reads from the 12 libraries to the 12x reference genome, in the BAM format and using the text alignment viewer of SAMtools [31]. Total RNA (1 μg) used for RNA-Seq was reverse-transcribed with iScript cDNA Synthesis Kit (BioRad) in a reaction volume of 20 μl. A 1/20 dilution was used as template for qPCR with iGreen qPCR Master Mix (Biomatik) on the CFX96 Real-Time system (BioRad) using the following conditions: 95°C for 20 s, followed by 39 cycles of 95°C for 3 s, 58°C for 25 s and 72°C for 25 s, followed by melt cycles from 55 to 95°C. Primer efficiency (E) was tested with dilutions of PCR products at values of 1.85 to 2 calculated as E = 10^(-1/slope)^. Primers gave a single PCR product, which was verified by gel electrophoresis and determination of melt curves at the end of each run. Differences in E, and between cycle thresholds (Ct) of the target (Ct^tar^) and the reference gene (Ct^ref^), were used to obtain normalized expression (N) of targets as N = E_tar_^Ct^tar^/E_ref_^Ct^ref^. *Ubiquitin* was selected as a reference transcript from a set of tested reference genes (S1 Table) [32], based on its consistently low coefficient of variation (CV) across biological replicates and treatments [range of 5 to 6%].

## Results

### Defining the latent phase of infection

From IW plants at 0.5 to 2 MPI, recovery of the pathogen was consistently high at the inoculation site, 0 cm (six of six plants, Table 1). In contrast, the first positive recovery beyond 0 cm was not until 1.5 MPI, albeit in only one of six IW plants, at both 2 cm above (2 cm) and 2 cm below (-2 cm) the inoculation site. It was not until 2 MPI that an increase in pathogen recovery at-2 cm in four of six plants signified significant spread of the pathogen. From 0.5 to 1.5 MPI, lesion lengths between IW and NIW plants were not significantly different. At 2 MPI, lesions were 10-fold larger than at 0.5 MPI, and 3-fold larger than at 1 and 1.5 MPI.

**Table 1:** Recovery of *N*. *parvum* was determined by culture, and fungal colonization of xylem fibers and rays was determined by light microscopy.

Treatment	Incubation period (Months post-inoculation, MPI)	Average lesion length (cm)	Recovery of *N. parvum* (No. plants out of 6 total)			Fungal colonization at 0 cm (*n* = 3 plants)	
			2 cm	0 cm	-2 cm	Median(% Stem area)	Relative treatment effect (95% Confidence limits)
**Inoculated—wounded (IW)**	0.5	0.3	0	6	0	47.42	0.71 (0.58, 0.84)
	1.0	1.0	0	6	0	38.47	0.71 (0.60, 0.82)
	1.5	1.0	1	6	1	60.90	0.85 (0.68, 1.01)
	2.0	3.0	1	6	4	60.37	0.85 (0.73, 0.96)
**Non-inoculated—wounded (NIW)**	0.5	0.3	0	0	0	10.95	0.29 (0.12, 0.46)
	1.0	0.3	0	0	0	14.43	0.41 (0.27, 0.56)
	1.5	0.3	0	0	0	9.98	0.25 (0.13, 0.38)
	2.0	0.4	0	0	0	11.25	0.34 (0.15, 0.53)
**Non-inoculated—non-wounded (NINW)**	0.0	—	0	0	0	5.16	0.09 (0.01, 0.18)

Each incubation period represents a separate set of plants inoculated at two-week intervals. Separate plants within each treatment—incubation period were used for recovery (six per treatment—incubation period) and microscopy (three per treatment—incubation period).

Recovery of *N*. *parvum* was negative from NIW and NINW plants at all time points and stem locations. These findings were supported by visual absence of the pathogen from NIW and NINW plants, specifically in the xylem fibers and rays in stem sections examined by light microscopy (Fig. 1 A). In contrast, these same xylem cells in IW plants were heavily colonized by the pathogen, especially at 0 cm (Fig. 1 B). Contaminating fungi (species of *Bionectria*, *Fusarium*, *Paecilomyces*) were recovered from all NIW plants at all time points from at least one stem location, reflecting the natural presence of these fungi as endophytes in the wood. From NINW plants, the same species of contaminating fungi were recovered from a total of three plants, at 1 and 2 MPI. The pathogen did not form fruiting structures on any IW plants and thus there was minimal risk of spores contaminating leaves with pathogen nucleic acids.

At 0 cm and at all incubation periods in IW plants, the pathogen was present in all six cell types/tissues (xylem fibers, xylem vessels, xylem rays, phloem, periderm, pith), based on qualitative observations of the presence of its pigmented hyphae, but to varying degrees depending on location. Beyond 0 cm there was visibly less colonization, which was restricted primarily to xylem fibers (at 2 cm) or both fibers and rays (at-2 cm). Based on the fact that fungal colonization was relatively sparse at 2 and -2 cm, and that the reaction zone was restricted to 0 cm, we focused on 0 cm for the labor-intensive measurements of fungal colonization (Fig. 1 B) and starch content in xylem fibers and rays (Fig. 1 C to G). In contrast, vessel occlusions were apparent at 2, 0, and -2 cm, so quantitation of tyloses and gels was carried out at all locations.

#### Infection response

**Fig 2:**
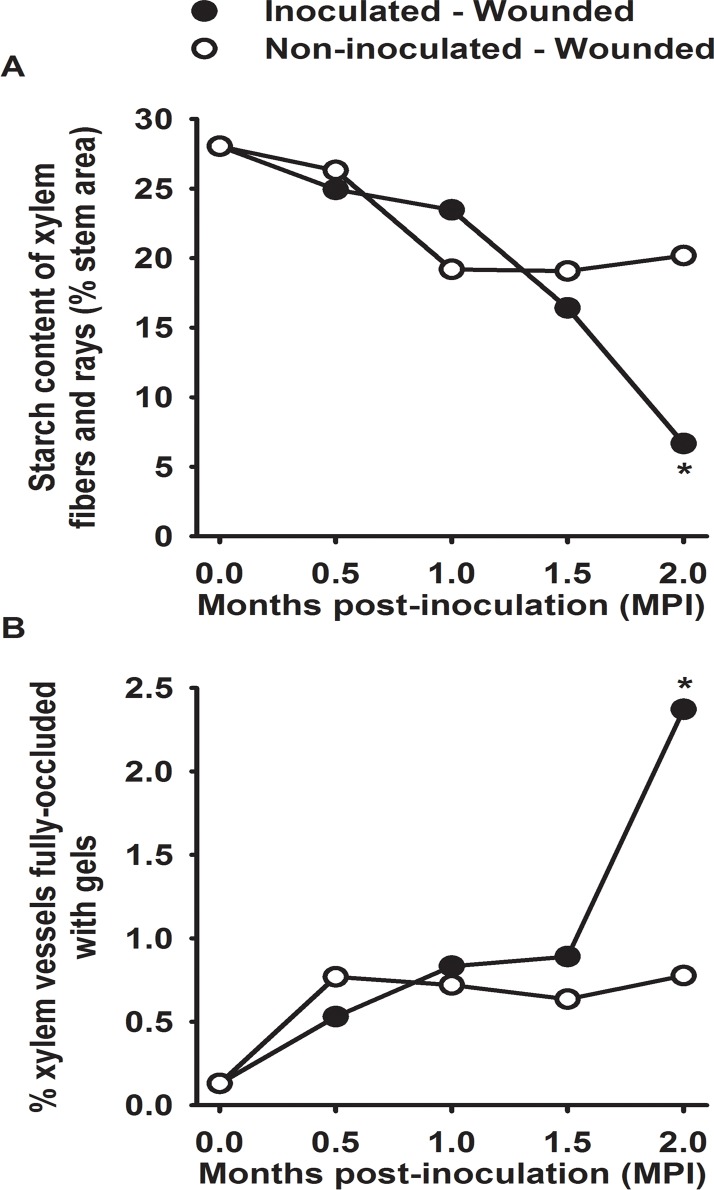
Plant responses to infection were significant at 2 MPI. (A) Starch content of the xylem fibers and rays decreased significantly in IW plants. Each point is the mean of three plants from stem sections at 0 cm. (B) Percentage of xylem vessels fully-occluded by gels increased significantly in IW plants. Each point is the mean of three plants, averaged across all three locations. The asterisk represents significant differences between IW and NIW plants; there were no significant differences in incubation periods without an asterisk (*P* ≤0.05, Tukey’s test; Table 2). NINW plants represent the 0 MPI incubation period (*n* = 3).

Based on Analyses of Variance (ANOVAs) of our quantitative observations, fungal colonization in xylem fibers and rays at 0 cm was significantly higher in IW versus NIW plants at all four incubation periods (*P* <0.0001, Table 1). At 2 MPI in IW plants, the increased frequency of pathogen recovery at-2 cm coincided with significant anatomical changes in the xylem. In IW plants, there was a decrease in starch content in xylem fibers and rays (Figs. 1 and 2 A) and a concomitant increase in the percentage of xylem vessels fully-occluded by gels (Fig. 2 B). In IW plants, starch content of xylem fibers and rays at 0 cm decreased significantly at 2 MPI (6.7% stem area, *n* = 3 plants), but remained at the same level in NIW plants over time (20.2% stem area, *n* = 3 plants; treatment-time effect of *P* = 0.0002). In IW plants, full gels increased significantly at 2 MPI (2.3% vessels fully-occluded by gels; *n* = 3 plants, averaged across three stem locations per plant), but remained at the same low levels in NIW plants over time (0.6% vessels fully-occluded by gels; *n* = 3 plants, averaged across three stem locations per plant; treatment-time effect of *P* = 0.0012; Table 2). Our finding of more vessels fully-occluded by gels in IW plants was consistent at all three stem locations, with the highest levels at 0 cm and the lowest at 2 cm (significant location effect of *P* = 0.0005, Table 2).

**Table 2:** ANOVAs of effects of nine treatment-incubation period combinations (NINW-0 MPI, IW-0.5 MPI, IW-1 MPI, IW-1.5 MPI, IW-2 MPI, NIW-0.5 MPI, NIW-1 MPI, NIW-1.5 MPI, NIW-2 MPI), stem location (2, 0, -2 cm), and their interaction on the percentages of xylem vessels partially or fully-occluded by tyloses or gels.

Effect	Num DF^a^	Den DF^b^	*F* values^c^			
			Partial tyloses	Partial gels ^0.5^	Full tyloses ^0.5^	Full gels ^0.5^
**Location**	2	36	8.76**	32.33***	23.25***	8.67**
**Treatment-incubation period**	8	18	1.35	1.81	1.60	3.84**
**Treatment- incubation period × Location**	16	36	2.60*	1.85*	1.66	0.90

^a^Num DF = Numerator degrees of freedom

^b^Den DF = Denominator degrees of freedom.

^c^*, **, and ** denotes significance at *P* = 0.05, *P* = 0.001, and *P* < 0.0001, respectively.

**Fig 3:**
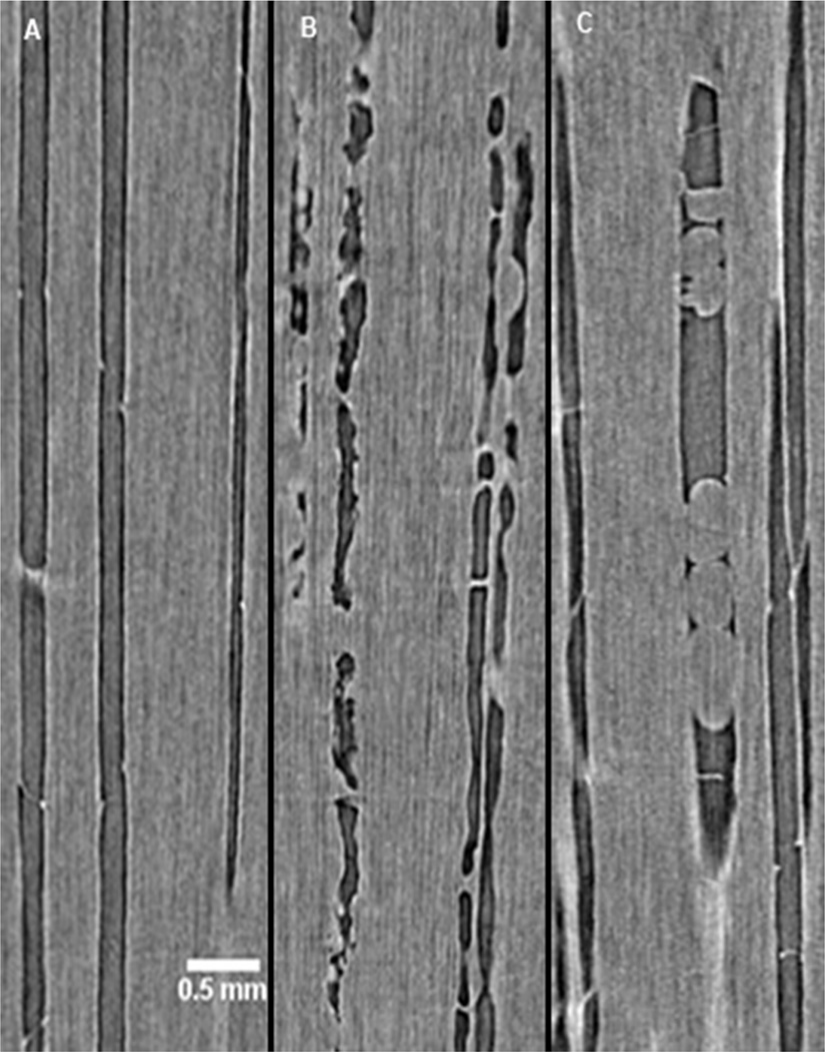
HRCT distinguished gel and tyloses-occluded xylem vessels. Shown here are optical sections of plants representative of three treatment-time combinations. (A) NINW-0 MPI, with unoccluded vessels. (B) IW-2 MPI, with gel-occluded vessels. (C) NIW-2 MPI, with tyloses-occluded vessels. Three-dimensional projections from which these optical sections originated are shown in S1 Video.

HRCT revealed differences in xylem vessel occlusions at-1 cm, with differences primarily associated with infection, similar to microscopy. NINW plants had the highest proportion of unoccluded, embolized vessels, with uniformly-straight walls and empty lumens (Fig. 3 A, S1 Video). IW plants had the highest proportion of vessels with lumens lined with amorphous (*i*.*e*., no distinct edges) occlusions, which increased in frequency from 0.5 (S2 Video) to 2 MPI (Fig. 3 B, S3 Video). By comparing these occlusions with sections of the-1 cm location observed by light microscopy, it was apparent that the amorphous occlusions in the HRCT images were gels, which were indeed most frequent in microscopic examination of IW plants (Fig. 2 B) and have not been seen in our other experiments involving HRCT of grape (e.g., [16]). Embolized vessels in NIW plants were rarely gel-occluded, but were instead occluded by tyloses (balloon-shaped structures) (Fig. 3 C), which were present at similar levels at 0.5 MPI (S4 Video) and at 2 MPI (S5 Video). HRCT showed that all plants, regardless of inoculation treatment or incubation period, had embolized vessels near the pith, which likely formed during plant propagation, when the xylem was cut and thus exposed to atmospheric pressure. The three-dimensional perspective gained by HRCT revealed vessel dysfunction at much higher rates (*c*. 4x greater than light microscopy) because of the ability to inspect vessel anatomy over a greater axial distance.

#### Wound response

Levels of xylem vessels that were partially-occluded by tyloses, as quantified by microscopy, were not significantly different in IW versus NIW plants (*P* = 0.3, Table 2), suggesting that this type of occlusion is a wound response. In both IW and NIW plants, partially-occluded vessels were most pronounced at 0.5 MPI at 0 cm (*data not shown*), hence the significant treatment-time × location effects on partial tyloses (*P* = 0.0086, Table 2). Levels of vessels that were fully occluded by tyloses were steady through all time points, and were highest at 0 cm in both IW and NIW plants (6.34% vessels fully-occluded by tyloses; *n* = 3 observations, averaged across both inoculation treatments and all four incubation periods).

### Differentially-expressed genes (DEGs) detected by RNA-Seq at 0 to 1.5 MPI

**Fig 4:**
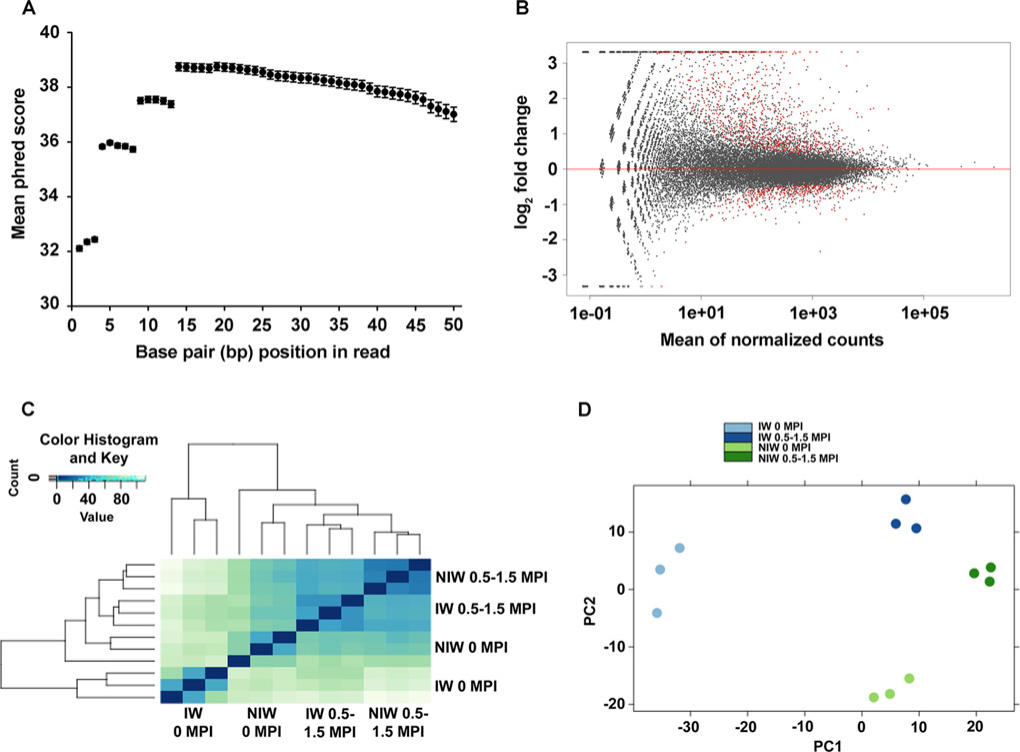
RNA-Seq revealed transcriptional differences in leaves of the four treatment-time combinations. (A) Per-base quality score plot shows the average Phred+33 score of all 12 samples. Error bars represent standard deviations. (B) Scatter plot showing differential expression for the contrast IW vs. NIW at 10% FDR. Red-coloured dots represent DEGs. Those positioned farthest from the x-axis, at the upper and lower plot borders, have very large to infinite log_2_-fold changes. (C) Heatmap showing Euclidean distances among the 12 libraries, calculated from Variance stabilized transformed (VST) count data. Colour key indicates level of similarity between libraries. (D) Principal Component Analysis (PCA) of the first two principal components (PC1, PC2).

Sequencing using HiSeq 2500 technology generated a total of 2.8 x 10^8^ single-end 50-bp reads with *c*. 1.4 x 10^8^ reads for NIW and IW libraries, respectively. On average, 24-million reads were generated per library. Quality scores across individual nucleotide bases were consistently high in all biological replicates (Fig. 4 A). No quality filtering, aside from trimming of the initial 13 bp, was applied and mapping statistics were used to compare trimmed and untrimmed datasets. Using this strategy, 93% (± 0.55 SD) of untrimmed reads mapped to the genome and 7.7% (± 0.24 SD) of these exhibited multiple alignments. In comparison, trimming led to a slight increase in mapped (95%, ± 0.40 SD) and also in multiple-mapped reads (10.1% ± 0.36 SD). For downstream analysis, only original untrimmed reads were used.

To determine whether the same transcripts were expressed in similar strengths in IW and NIW libraries, read count distributions for individual genes were compared. In both IW and NIW plants, *c*. 14% of transcripts were predicted to not be expressed. In IW plants, 26,079 transcripts were expressed, compared to 25,749 in NIW plants, with an overlay of 25,249 genes with comparable read counts. Therefore, IW and NIW samples were comparable. In IW plants, 830 genes were expressed, which were not expressed in NIW plants (S2 Table). As these 830 IW-specific genes may include putative candidate genes with low expression levels, and hence low read counts, their characterization as differentially expressed can be hampered by Poisson (*a*.*k*.*a*. ‘shot’) noise [27]. To account for this, the minimum number of total read counts was estimated as 10 by plotting the mean of normalized read counts against the log_2_-fold change for the contrast of IW versus NIW (Fig. 4 B). Principal Component Analysis (PCA) of Euclidean distances indicated that biological replicates grouped together, suggesting that gene expression was significantly influenced by the combined effects of incubation period (0 MPI vs 0.5–1.5 MPI) and treatment (IW vs. NIW), and not by variation among replicates (Fig. 4 C and D).

Using DESeq, 2,130 DEGs (*c*. 7% of total predicted transcripts, S3 Table) were identified between IW and NIW plants, including 11 of the 830 IW-specific genes (S2 Table). With a log_2_-fold change >1, we found 633 genes were significantly up-regulated and 55 were significantly down-regulated (S3 Table). Analysis for Gene Ontology (GO) functional category enrichment in the 633 induced DEGs revealed that, within the term ‘Biological Process’, the most abundant categories were response to water deprivation, ATP synthesis, coupled proton transport, and cellular respiration (S4 Table). Analysis for the main category ‘Molecular Function’ showed that 8.2% of induced DEGs were linked to subcategories of ‘binding’ (e.g., iron ion, heme, chitin). Oxidoreductase activities (e.g., monooxygenase, catalase, dehydrogenase), were well-represented catalytic activities among induced DEGs (S4 Table). No GO categories were significantly over-represented in the 55 repressed DEGs (*data not shown*).

#### Selection of candidate genes through screening of publicly available datasets and qPCR analysis

To select candidate genes, we arranged the 633 induced DEGs into six clusters, based on their expression pattern in the four time-treatment conditions (S1 Fig.). Candidates were then chosen from clusters 5 and 6, which showed higher expression in IW versus NIW plants and persistence over incubation periods 0 MPI and 0.5–1.5 MPI, in contrast to genes from clusters 1 to 4. As the IW-0 MPI and NIW-0 MPI leaves were sampled 10 min after inoculation, the elevated transcript abundance represented in clusters 5 and 6 indicated a very rapid response in the leaves to the presence of the pathogen in the stem.

Further data reduction was performed by selection of 20 candidate genes from clusters 5 and 6, which were not transcriptionally responsive to a range of common vineyard pathogens, pests, or abiotic stresses, as examined in 21 studies for which microarray datasets are publicly available (Table 3, S5 Table). Candidate genes (Table 3) were non-responsive to the following: trunk disease Eutypa dieback (fungal pathogen *Eutypa lata*), foliar diseases powdery mildew (fungal pathogen *Erysiphe necator*) and downy mildew (oomycete pathogen *Plasmopara viticola*), vascular diseases caused by prokaryotes [Bois noir (phytoplasma pathogen stolbur) and Pierce’s disease (bacterial pathogen *Xylella fastidiosa*)], vine mealybug (causal insect *Planococcus ficus*), and heat and UV light stresses (S5 Table).

**Table 3:** Top 20 DEGs as a result of inoculation treatment and incubation period.

Gene stable ID	UniProt ID	Short annotation	Log2 ratio^a^			Differentially-expressed in publicly available datasets^c^
			0.5 to 1.5 MPI vs. 0 MPI	NIW vs. IW	P value^b^	
**VIT_08s0007g06430**	A5BCB2	Late embryogenesis abundant 4–5	-0.47	-32.17	3.15E-03	2/11
**VIT_04s0023g02480**	A5C8L5	Dehydrin	0.88	-7.09	7.21E-11	9/20
**VIT_05s0020g00330**	F6HE08	Galactinol synthase 2	-0.08	-6.05	8.47E-06	8/21
**VIT_14s0006g01580**	D7TSS2	Unknown	3.05	-5.57	9.92E-03	7/20
**VIT_05s0049g02240**	D7SZQ8	AWPM-19-like family protein	0.18	-5.2	6.14E-09	8/21
**VIT_08s0007g04240**	F6HKF4	Embryonic cell protein 63	2.7	-5.08	1.98E-07	6/21
**VIT_06s0004g06830**	G3G8K0	Glutamine-dependent asparagine synthase 1	0.63	-4.09	3.33E-05	7/20
**VIT_05s0029g00370**	F6GWE8	Phytocystatin 2	-1.47	-3.91	7.81E-03	4/18
**VIT_14s0066g00700**	A5ALS9	Oleosin family protein	0.62	-3.78	3.09E-03	4/20
**VIT_07s0151g00640**	F6HI56	Cupin family protein	-1.24	-3.33	4.74E-06	2/11
**VIT_11s0016g03950**	F6HH21	BURP domain-containing protein	0.63	-3.19	4.07E-04	0/21
**VIT_12s0057g00120**	F6HHJ0	Wound-responsive family protein	-0.45	-2.71	6.80E-06	7/18
**VIT_12s0059g00470**	F6HIA6	Unknown	1.69	-2.61	4.22E-03	0/19
**VIT_14s0083g01140**	F6GVV3	B12D protein	-0.62	-2.49	7.79E-07	5/20
**VIT_19s0014g03290**	E0CSQ1	NAC domain containing protein 19	0.35	-2.47	1.58E-10	7/20
**VIT_12s0057g00090**	F6HHI7	Wound-responsive family protein	-0.75	-2.33	4.76E-14	6/20
**VIT_08s0007g01400**	A5BVL9	Glutathione S-transferase TAU 8	-1.41	-2.13	2.06E-04	2/11
**VIT_08s0007g05580**	D7TIL3	S-adenosyl-L-methionine-dependent methyltransferase	-0.05	-2.06	6.69E-04	8/21
**VIT_12s0059g00440**	D7TE59	Unknown	-0.25	-2.04	1.35E-08	6/18
**VIT_12s0057g00170**	F6HHJ4	Wound-responsive family protein	-0.49	-2.01	1.51E-05	6/20

For a complete list of fitted coefficents for all genes calculated via DESeq, see S3 Table.

^a^Columns show the fitted coefficients, converted to a log_2_ scale.

^b^Benjamini Hochberg adjusted *P* value.

^c^Number of published grapevine studies in which the gene was differentially expressed, out of a maximum of 21 publicly available microarrays datasets. As not all genes were represented on the three chip platforms in all 21 datasets, the denominator varies from 11 to 21 (details of each experiment are given in S5 Table).

**Fig 5:**
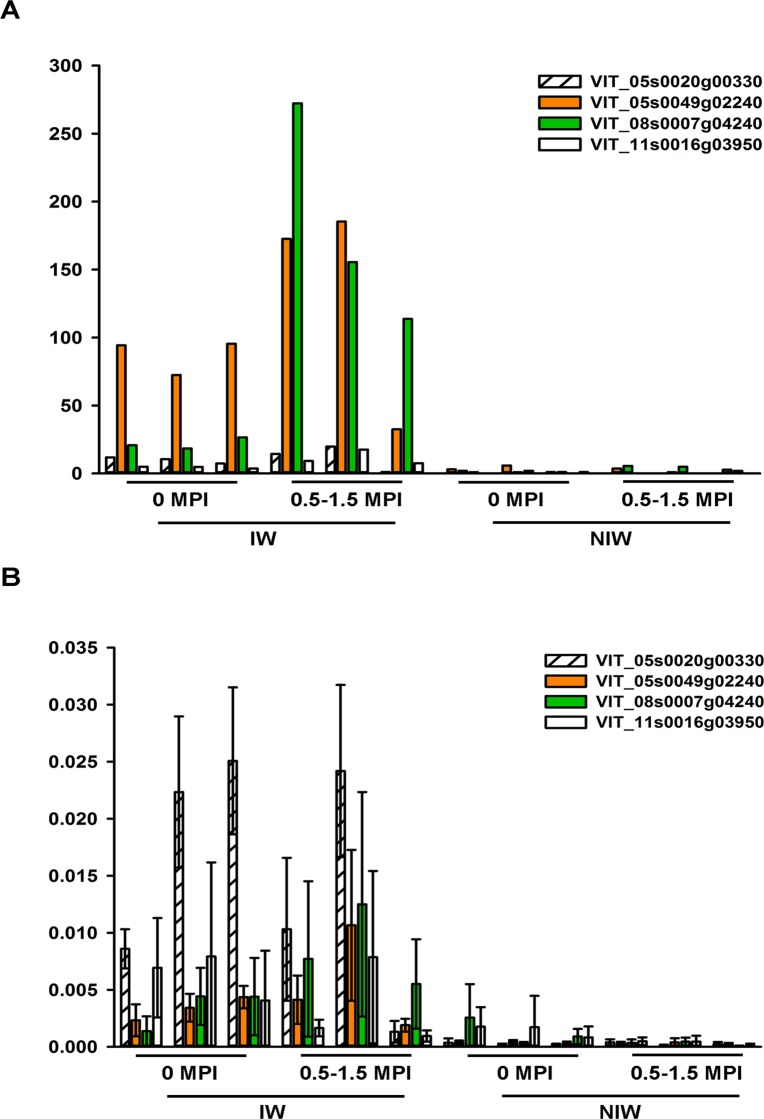
Differential expression analysis of four candidate genes: a galactinol synthase (VIT_05s0020g00330), an abscisic acid-induced wheat plasma membrane polypeptide 19 ortholog (VIT_05s0049g02240), an embryonic cell protein 63 (VIT_08s0007g04240), and a BURP domain-containing protein (VIT_11s0016g03950). (A) DESeq analysis of differential expression. Each column represents the normalized read counts from RNA-Seq of one replication. (B) qPCR validation of DESeq counts. Transcript levels of marker genes were corrected to *VvUbiquitin1* (TC32075). Each column is the mean of six replicate PCR reactions. Error bars represent standard errors.

RNA-Seq normalized count data showed changes in expression between IW and NIW, ranging from 1- to 170-fold. qPCR was performed on 13 of the 633 DEGs with a Spearman correlation coefficient of 0.9, verifying the reliability and dynamic range of RNA-Seq results (S2 Fig.). Elevated mRNA levels measured by qPCR correlated well with an increase in sequencing reads originating from candidate gene chromosomal locations, exemplarily shown for the four best candidates: a galactinol synthase (locus ID: VIT_05s0020g00330), an abscisic acid-induced wheat plasma membrane polypeptide 19 ortholog (AWPM-19-like, VIT_05s0049g02240), an embryonic cell protein 63 (VIT_08s0007g04240), and a BURP domain-containing protein [VIT_11s0016g03950 (Fig. 5, Table 3)].

## Discussion

The latent phase of grapevine infection by *N*. *parvum* could be defined as the point before the pathogen begins to spread from the inoculation site and before the host undergoes anatomical changes in response to infection. Under our experimental conditions, between 0 and 1.5 MPI, the series of events during the latent phase was as follows. Pathogen spread beyond the inoculation site was limited until 1.5 MPI and average lesion lengths were relatively stable. Also, there were no significant differences in xylem vessel occlusions over this time period. In spite of no significant changes in either infection development or wood anatomy, expression of 20 candidate genes, which were rapidly induced in the leaves within minutes of inoculation and persisted throughout the latent phase, brings new insight into the molecular response to infection. The large coverage of the host transcriptome through RNA-Seq (approx. 25,000 genes) provided the most thorough examination to date of a grapevine trunk disease. Screening of publicly-available transcriptome data from previous studies on grape, including those performed on the whole-genome NimbleGen microarray (GEO accession GPL13936, which represents 98.6% of genes predicted from the V1 annotation of the 12x genome) and two approximately half-genome Affymetrix microarray platforms (GEO accession GPL11004 and GEO accession GPL1320), showed that the four best candidates out of the 20 genes (a galactinol synthase, an AWPM-19-like protein, an embryonic cell protein 63, a BURP domain-containing protein) are not differentially regulated by development [33] or abiotic stresses [34, 35]. Furthermore, they are not differentially regulated by another common trunk disease, Eutypa dieback, or common diseases that attack grapevine leaves, powdery and downy mildews [12, 36].

Our findings of significant infection development from 1.5 to 2 MPI, coupled with anatomical changes in woody tissues/ cell types in response to the infection, but still no visible symptoms in the leaves, suggest that this period is the start of the pathogenic phase of *N*. *parvum*. From 1.5 to 2 MPI, lesion lengths increased significantly, which was concomitant with significantly higher levels of fungal colonization at the inoculation site and higher recovery rates beyond the inoculation site. Patterns of gel-occluded vessels mirrored fungal colonization, and both were concentrated at the inoculation site and to a lesser extent at-2 cm. Nonetheless, the gel-occluded vessels were a futile attempt by the plant (the susceptible cultivar ‘Cabernet Sauvignon’) at compartmentalizing *N*. *parvum* infection. As the pathogen spread beyond the inoculation site (mainly to -2 cm) it preferentially colonized xylem fibers and rays, and starch content in these cell types decreased to their lowest levels at 2 MPI.

It is difficult to predict how our findings from this greenhouse experiment relate to the infection response in the field. We inoculated wounded plants in July and August, but infection is thought to occur primarily during the dormant season [37]. Furthermore, grapevine xylem occlusions vary by season, with gels forming primarily in winter and tyloses in summer [21]. That said, we can at least compare the *N*. *parvum* infection process as we describe it here to that of a previous greenhouse study of *P*. *chlamydospora* [15] and a field study of *E*. *lata* [14], which are also trunk pathogens that infect primarily through pruning wounds during dormancy. Our finding that *N*. *parvum* colonized all woody tissues/cell types initially, but later spread through xylem fibers and rays is consistent with the behavior of *P*. *chlamydospora*, albeit based on their more extensive microscopic observations of fungal colonization and wood anatomy/ biochemistry [15]. We found that levels and distributions of tyloses did not vary between IW and NIW plants, suggesting that tyloses are a wound response. Indeed, the highest levels of tyloses were at 0 cm, regardless of inoculation treatment, which may reflect the plant’s attempt to prevent water loss from vessels damaged at the wound site, as is hypothesized by Pouzoulet et al., who also found tyloses to be a wound response to *P*. *chlamydospora* [15]. Decreasing starch content of the xylem fibers and rays in response to *N*. *parvum* is also reported for *E*. *lata* [14]. Starch depletion is thought to result from enzymatic degradation of the xylem fibers and rays by *E*. *lata* [38], which has also been shown for another soft rot fungus *Ophiostoma novo-ulmi*, causal agent of Dutch elm disease [39]. *N*. *parvum* has not been characterized as a soft rot fungus, although extracellular compounds from pure cultures cause necrosis of grapevine wood [40] and the genome of the pathogen codes for numerous cell wall-degrading enzymes [41].

Although discussion of biological functions based on the transcript profiles is preliminary, we hypothesize that several genes from clusters 1 to 4, which encompasses ascorbate peroxidases, glutathione S-transferases (GSTs), and catalases, are upregulated as part of a general stress response in leaves, which correlates well with changes in levels of the same enzymes by *N*. *parvum* in symptomatic wood of vines with Botryosphaeria dieback [42]. Clusters 1 to 4 also contained putative amylases and plant invertases, which are involved in carbohydrate metabolism. Enhanced expression and activity of cell wall invertases is reported in several plant—pathogen interactions [43]. Their induction in leaves during the latent phase of infection might be associated with the subsequent starch depletion we detected in xylem fibers and rays in the pathogenic phase; this is an alternative hypothesis to enzymatic degradation of these cell types by *N*. *parvum*. Although we did not measure starch levels of the entire developing lesion, the shift in carbon usage at the inoculation site suggests that the developing canker might provide a new competing sink tissue, which may accumulate sugars, as has been shown during formation of haustoria by the rust fungus *Uromyces fabae* on broad bean [44]. Alternatively, canker development uses up starch from nearby cellular structures by catabolism. As this would interfere with the source-sink balance in the host, young leaves may be strengthened as sink tissue in order to sustain normal plant growth by maintaining high hexose availability and decreasing export of assimilates by upregulation of plant invertases. Indeed, eight putative invertases were significantly upregulated in IW plants and may contribute to high invertase activity, as shown previously for grapevines infected with the fruit and foliar pathogen *Botrytis cinerea* [45].

Our examination of general trends in expression levels in IW plants showed that enrichment of GO terms is dominated by the molecular functions iron ion and heme binding, and oxidoreductase activities, which are well represented in clusters 1 to 4. However, their induction in leaves during the latent phase of infection suggests that there might be an antecedent response to drought/oxidative stress, associated with vascular system damage by the pathogen and/or the plant’s attempts to compartmentalize it. In Arabidopsis, transcript profiles revealed that changes induced by several pathogens, albeit with different modes of attack, can nonetheless show considerable overlap, especially with respect to stress-related genes [46]. Taxonomically-unrelated fungal pathogens *Phytophthora infestans* and *Cladosporium fulvum* trigger production of reactive oxygen species (ROS) by plant NADH:ubiquinone oxidoreductases [47, 48], which were also significantly upregulated in our IW plants.

In contrast to clusters 1 to 4, genes in clusters 5 and 6 did not show any overrepresentation of GO terms and might therefore contain a more complex, *N*. *parvum*-specific response signature. Analysis of the spatio-temporal expression pattern of all 110 genes from clusters 5 and 6, using the grapevine Gene Atlas [33], showed that most candidate genes were not or only weakly expressed in younger leaf stages when compared to senescent leaves or other tissues. This was confirmed in our study using RNA-Seq and qPCR on NIW samples. As such, we hypothesize they are not regulated during leaf development to fulfill biological functions and therefore might serve as host-based markers for reliable detection of *N*. *parvum* infection. More experiments are needed to dissect the transcriptomic response of potential markers for *N*. *parvum* from the response to co-ocurring and changing environmental cues, such as dehydration and other pathogens.

## Conclusions

A detection tool for the early stage of infection might encourage adoption of disease management practices in newly-established vineyards, before Botryosphaeria dieback becomes widespread. It also has utility for detecting infected mother vines in the nursery and for preventing the subsequent commercial dissemination of asymptomatic, infected plant material. Cumulative crop losses from the chronic infections of *N*. *parvum* and other trunk diseases build up in California vineyards to levels of *c*. 70% by the 15^th^ year [49], at which point annual production costs outweigh returns [50]. As such, the profitable lifespan of a vineyard with Botryosphaeria dieback and other trunk diseases is typically only 15 to 20 years. Effective disease management relies on preventative measures [51–53]. It is typically not until symptoms become widespread in the vineyard—on at least 20% of the vines in years 8 to 10 [49]—that farmers are alerted to the problem. At this point, however, adopting preventative practices is of little use.

Further experiments are needed to determine whether the candidate genes are truly specific to *N*. *parvum*-caused Botryosphaeria dieback of grapevine. For example, a similar approach using RNA-Seq and complementary qPCR is needed on other grapevine cultivars infected by *N*. *parvum*, and to test for possible cross-reactivity with foliar pathogens, other trunk diseases (e.g., Eutypa dieback), or drought stress. In addition to detection, these markers, if found to be specific in the field, can have other applications to farming; they may help reveal that a change in grapevine physiology, due to an abiotic stress for example, shortens the latent phase. In turn, knowledge of the abiotic stress trigger, especially in the case of inducing the pathogenic phase, can thus lead to new management practices that do not promote disease.

## Supporting Information

S1 Fig Cluster analyses of genes partitioned into six clusters. The R package Hopach was used to partition genes with similar expression patterns into clusters, using the distance metric ‘cosangle’ for calculating dissimilarities between variables. Membership of a gene to a cluster is based on resampling performed with 100 non-parametric bootstraps.
(TIF)

S2 Fig Correlation of qPCR (fold change) and RNA-Seq (counts fold change) data for 13 candidate genes (*P* <0.001). VIT_00s1455g00010, VIT_01s0026g02710, VIT_04s0023g02480, VIT_05s0020g00330, VIT_05s0049g02240, VIT_06s0004g06830, VIT_06s0009g02780, VIT_07s0005g05600, VIT_08s0007g04240, VIT_09s0002g06790, VIT_11s0016g03950, VIT_16s0115g00170, VIT_18s0001g00140 (for a complete list of fitted coefficents for these genes, calculated via DESeq, see S3 Table). Each point represents the mean of six replicate qPCR reactions plotted against the mean of six IW: NIW counts from six RNA-Seq samples, per treatment-time combination. Spearman’s rho = 0.9.
(TIF)

S1 Table Primers used for qPCR and PCR approaches. (XLSX)

S2 Table Genes expressed in IW samples, based solely on read counting. (XLSX)

S3 Table Complete list of DEGs, due to *N*. *parvum* infection, and their partition in six clusters based on expression in IW and NIW samples. (XLSX)

S4 Table GO analysis of differential-expressed genes with log2 fold change > 1. (XLSX)

S5 Table Expression analysis of 110 candidate genes from clusters 5 and 6, in 21 publicly available grapevine transcriptome data. (XLSX)

S1 Video HRCT of NINW plant at 0 MPI. The video displays a series of tangential, optical HRCT scans of the stem of a living, potted grape plant. The series moves horizontally, starting at the pith and moving out towards the bark. The black columns that appear and disappear as the video plays are embolized xylem vessels, which are filled with air instead of water, and are thus non-functional. The more centrally-located, segmented columns are the pith. NINW plants had primarily unoccluded, embolized vessels, with uniformly-straight walls and empty lumens.
(MOV)

S2 Video HRCT of IW plant at 0.5 MPI. Starting at 0.5 MPI and continuing throughout all incubation periods, IW plants had consistent levels of vessels occluded by tyloses, which appear as balloon-shaped structures (of various sizes) with well-defined edges, within the lumens of embolized vessels.
(MOV)

S3 Video HRCT of IW plant at 2 MPI. Over time, IW plants had increasing levels of vessels occluded by gels, which appear as an amorphous lining against the vessel wall that visibly constricts the lumen. Gels were unique to IW plants.
(MOV)

S4 Video HRCT of NIW plant at 0.5 MPI. Throughout the course of the experiment, NIW plants had consistent levels of vessels occluded by tyloses, which appear as balloon-shaped structures (of various sizes) with well-defined edges, within the lumens of embolized vessels.
(MOV)

S5 Video HRCT of NIW plant at 2 MPI. Over time, NIW plants had consistent levels of vessels occluded by tyloses. Because the frequencies of tyloses-occluded vessels were not significantly different between IW and NIW plants, or among incubation periods, the formation of tyloses is likely a wound response, as both IW and NIW plants were wounded.
(MOV)
