# Supplementary figures and images for: Genes Expressed in Grapevine Leaves Reveal Latent Wood Infection by the Fungal Pathogen Neofusicoccum parvum
Source: PLoS One. 2015 Mar 23;10(3):e0121828. doi: 10.1371/journal.pone.0121828 (PMC4370485; doi:10.1371/journal.pone.0121828)

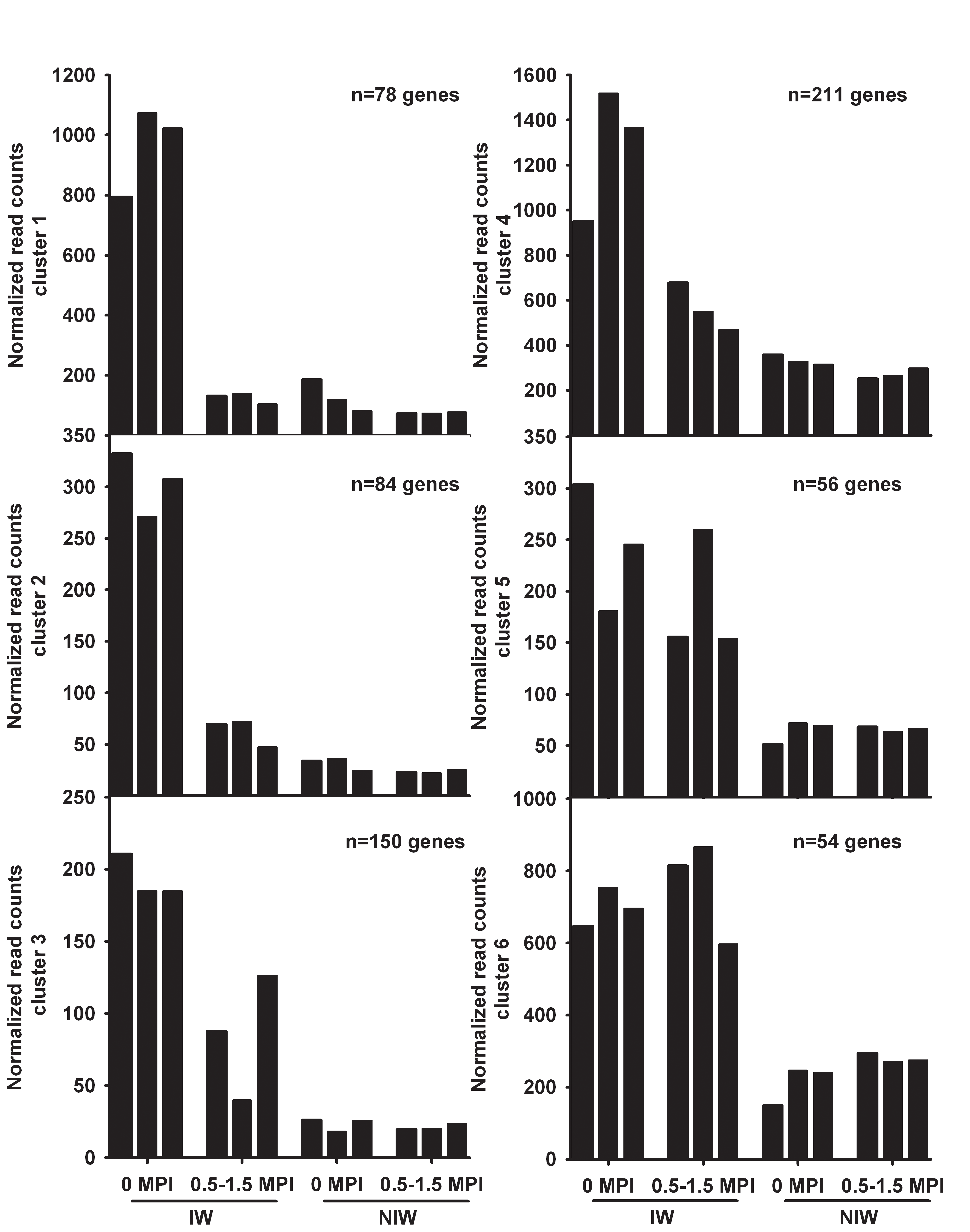

Supplement: S1 Fig — The R package Hopach was used to partition genes with similar expression patterns into clusters, using the distance metric ‘cosangle’ for calculating dissimilarities between variables. Membership of a gene to a cluster is based on resampling performed with 100 non-parametric bootstraps. (TIF) [file pone.0121828.s001.TIF]

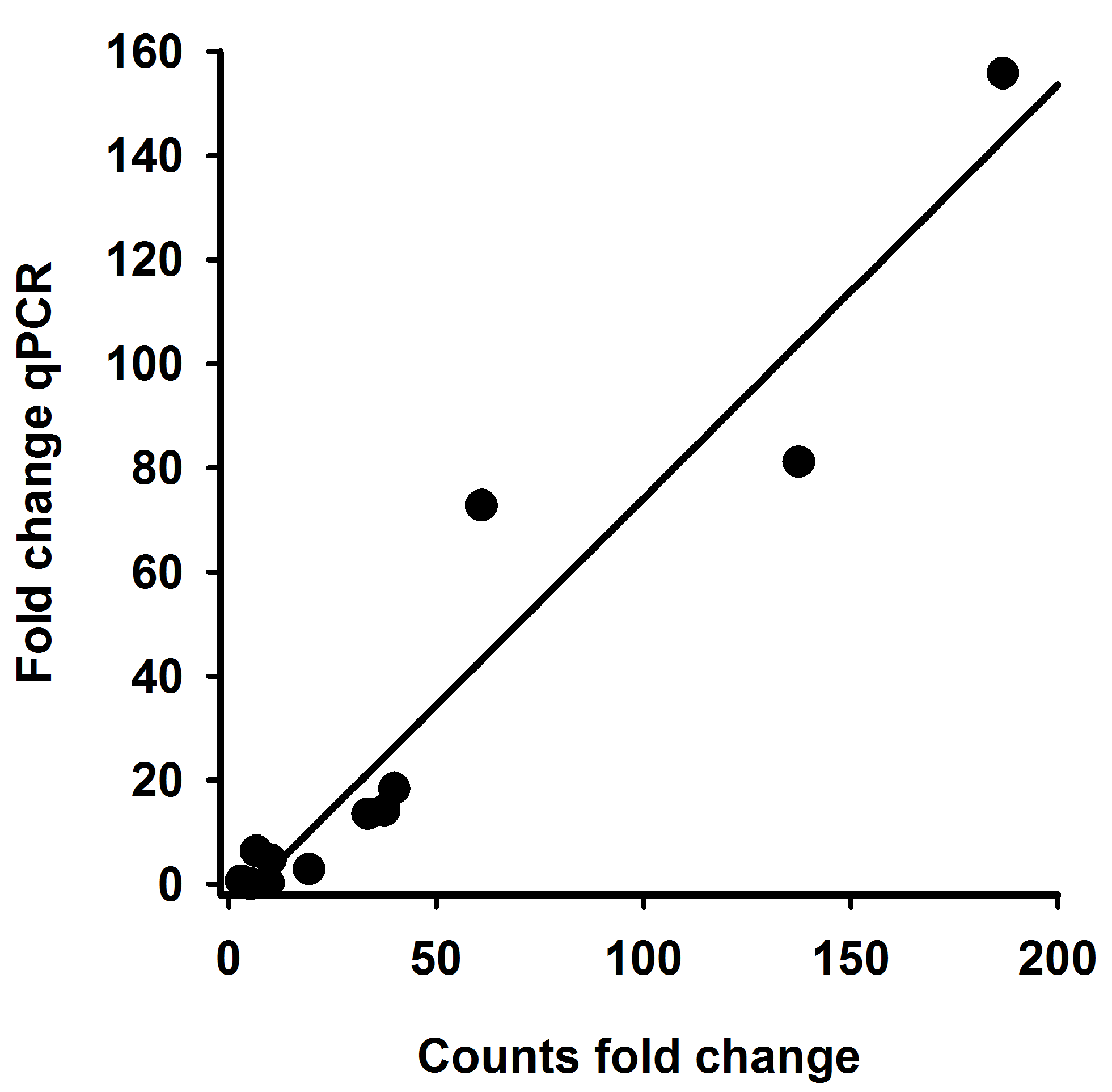

Supplement: S2 Fig — VIT_00s1455g00010, VIT_01s0026g02710, VIT_04s0023g02480, VIT_05s0020g00330, VIT_05s0049g02240, VIT_06s0004g06830, VIT_06s0009g02780, VIT_07s0005g05600, VIT_08s0007g04240, VIT_09s0002g06790, VIT_11s0016g03950, VIT_16s0115g00170, VIT_18s0001g00140 (for a complete list of fitted coefficents for these genes, calculated via DESeq, see S3 Table). Each point represents the mean of six replicate qPCR reactions plotted against the mean of six IW: NIW counts from six RNA-Seq samples, per treatment-time combination. Spearman’s rho = 0.9. (TIF) [file pone.0121828.s002.tif]
